# Combination of triple chemotherapy and sequential re-irradiation as salvage for recurrent treatment-refractory hemangiopericytoma of extraspinal dura: a case report

**DOI:** 10.3389/fonc.2024.1405755

**Published:** 2024-11-22

**Authors:** Min Wang, Wanrui Lv, Xi Chen, Ke Cheng

**Affiliations:** ^1^ Division of Radiotherapy, Cancer Center, West China Hospital, Sichuan University, Chengdu, China; ^2^ Department of Oncology, Meishan City People’s Hospital, Meishan, Sichuan, China; ^3^ Division of Abdominal Tumor Multimodality Treatment, Cancer Center, West China Hospital, Sichuan University, Chengdu, Sichuan, China

**Keywords:** hemangiopericytoma, solitary fibrous tumor, re-irradiation, triple chemotherapy, HPC

## Abstract

**Introduction:**

Hemangiopericytoma (HPC) in the central nervous system (CNS) is rare. Our report aims to present an HPC case with multiple surgeries at the lumbar spine, and demonstrates an effective treatment as salvage.

**Case report:**

In this report, we present the case of a young girl with recurrent meningeal invasion of lumbar spinal HPC. The patient underwent multiple surgeries to remove the tumors, and adjuvant radiotherapy was administered after the initial resection. And it presented a pathological anaplastic transformation with subsequent accelerated recurrence. A combination therapy approach involving triple chemotherapy and sequential re-irradiation was found to be effective as salvage treatment at the third recurrence.

**Conclusions:**

Surgical resection remains the primary treatment modality for HPC in the spine, despite its high tendency for local recurrence and the risk of metastasis. For unresectable recurrent HPC, combining chemotherapy and sequential re-irradiation might be a highly effective, and safe reference regimen as the salvage treatment for the refractory case.

## Introduction

Hemangiopericytoma (HPC), a relatively rare soft tissue vascular tumor, originates from Zimmermann pericytes, the contractile cells surrounding capillaries and venules. Commonly found in the retroperitoneum, extremities, and head and neck area (1), HPC rarely occurs in the CNS, including the skull base, spinal canal, and meninges, as reported in limited studies (2, 3). Intracranial HPC is widely recognized as an aggressive disease with high recurrence rates (4).

Tumors originating within the spinal canal, whether primary or metastatic, often lead to spinal cord compression, causing a range of CNS symptoms, including pain, motor weakness, sensory changes, sphincter dysfunctions, and potentially their simultaneous occurrence. Neurological compromise caused by HPC progresses rapidly, and delays in diagnosis and treatment can result in potentially irreversible neurological impairment, significantly diminishing patients’ survival and quality of life.

In this report, we present a rare case of primary intraspinal extramedullary HPC located in the lumbar spinal canal. It exhibited a pathological anaplastic transformation, leading to rapid recurrence. Despite multiple recurrences following surgery and adjuvant radiotherapy, the tumor responded positively to a combination of triple chemotherapy and sequential re-irradiation upon its third recurrence.

## Case report

**Figure 1:**
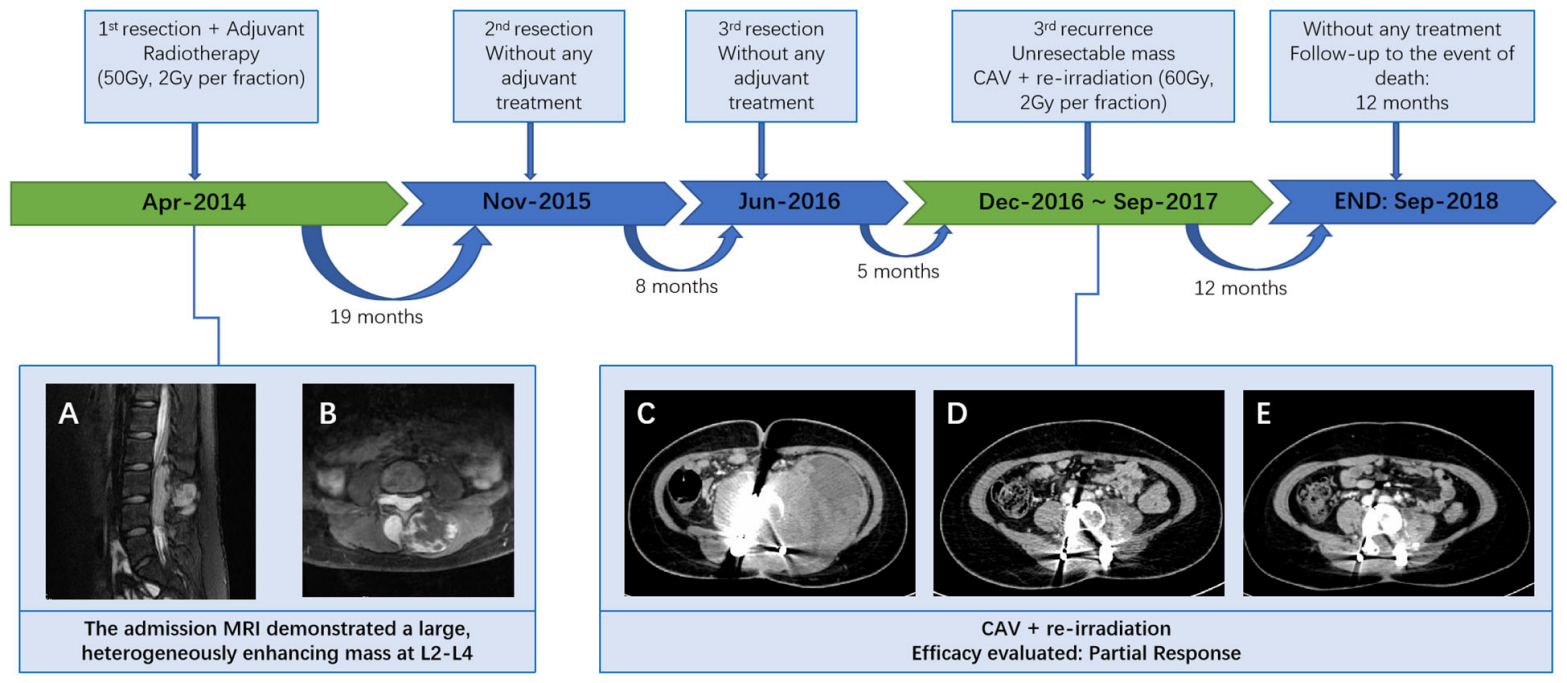
Patient treatment flow **(A, B)** MRI of the lumbar spine demonstrated a lesion at the L2-4 level, which invaded both the intraspinal extramedullary dura and the extraspinal canal tissue. **(C)** At the third recurrence, the CT scan demonstrated a giant, unevenly dense mass shadow at L3-L5 and left retroperitoneum; **(D)** following radiotherapy and 4 cycles of CAV, demonstrating a deep shrinkage of the mass; **(E)** following 8 cycles of CAV, indicating a further shrinkage.

**Figure 2:**
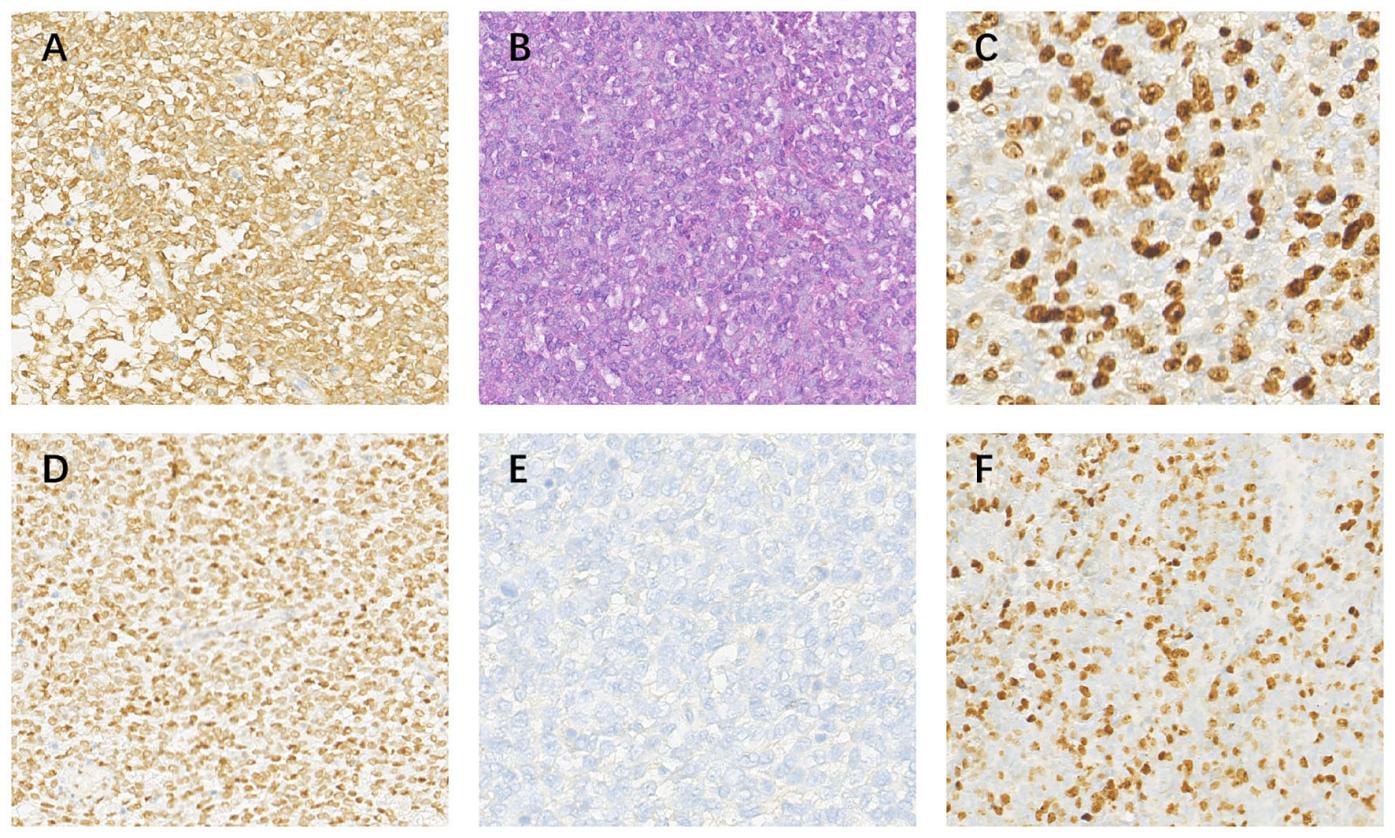
Pathological images at 20 x **(A)** BCL-2(+); **(B)** HE; **(C)** Ki-67(+,40%); **(D)**TLE-1(+); **(E)** EMA(-); **(F)** MIB(-).

**Figure 3:**
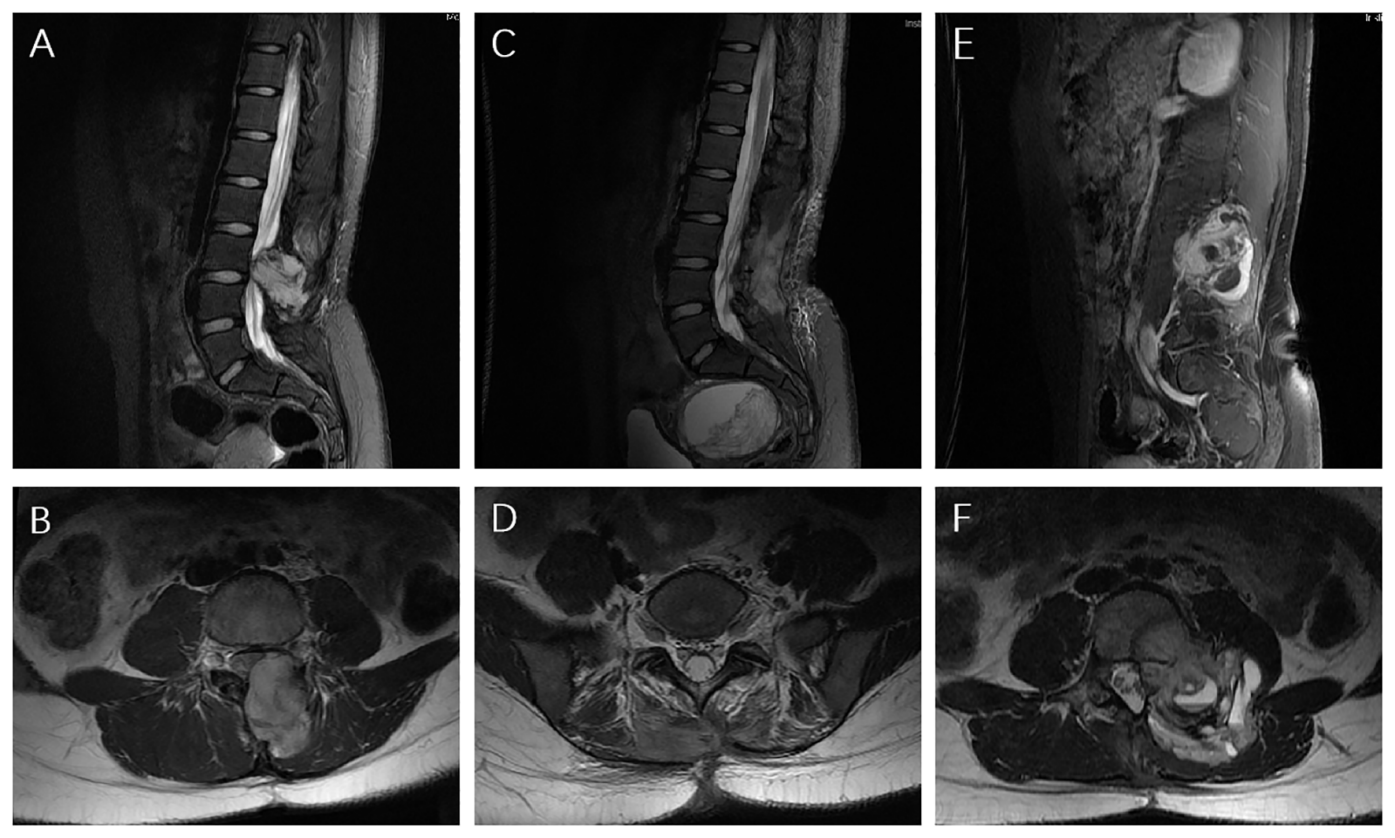
MRI images **(A, B)** At the first recurrence, MRI exhibited an unusual hyper-vascular mass; **(C, D)** Postoperative it showed no residual or occurrence of the tumor; **(E, F)** 8 months later, it exhibited the second recurrence.

A 14-year-old girl was admitted to our hospital center in April 2014, and presented with a three-month history of lumbar back pain. MRI of the lumbar spine demonstrated a lesion at the L2-4 level, which invaded both the intraspinal extramedullary dura and the extraspinal canal tissue as depicted in **Figures 1A, B**. The mass exhibited a slightly hyperintense signal on T1WI and a pronounced hyperintense signal on T2WI, with significant heterogeneity observed in the enhancement pattern following contrast administration. A gross total resection (GTR) was performed, with an estimated blood loss of 100 mL, followed by adjuvant external beam radiotherapy (EBRT) with a total dose of 50Gy (2 Gy per fraction). Pathological analysis confirmed the diagnosis as HPC, WHO grade II, as assessed by two experienced pathologists. Immunohistochemical staining showed positive expression for Bcl-2, TLE, Vim, and Ki67 (nearly 40%), and negative expression for STAT-6, PCK, EMA, SMA, CD57, CD99, Des, and S-100 (**Figure 2**). Fluorescence *in situ* hybridization (FISH) revealed no rearrangement of the SS18 gene, and genetic testing did not reveal the fusion of EWSR1. After 19 months of regular follow-ups with no signs of local recurrence or metastasis, the patient was re-admitted in November 2015, with increasing lumbar back pain, bilateral motor weakness graded as 4 on the Medical Research Council (MRC) scale, and lower limb numbness. MRI revealed a possibility of recurrence (**Figures 3A, B**). A second resection (**Figures 3C, D**) was performed without adjuvant therapy. This intervention led to the absence of further neurological complications, and a complete resolution of the patient’s motor weakness and sensory disturbances. Pathological analysis of the resected tissue indicated a change in the tumor grade from WHO II to WHO III, suggesting pathological deterioration and a tendency toward increased aggressiveness.

In June 2016, following the identification of a recurrent mass on MRI (**Figures 3E, F**), the patient underwent an additional extensive resection procedure which included internal fixation with pedicle screws. Based on the surgeon’s assessment, the tumor was resectable, and the associated bone damage was minimal. Pedicle screw fixation was considered sufficient to ensure spinal stability, therefore, no further fusion methods, including bone grafting, were performed at the time. Unexpectedly, only 5 months later, the patient was re-admitted with severe lumbar back pain, and also reported difficulty with bowel and bladder function. Moreover, there was a bilateral reduction in skin sensation below the iliac crests, with the right side being more severely affected. The muscle strength of both lower limbs was graded as 2 on the MRC scale. Enhanced CT examination (**Figure 1C**) revealed an abnormally large, unevenly dense mass shadow at L3-L5 and the left retroperitoneum, causing serious damage to the adjacent vertebral body, rendering it unresectable. Due to the high burden of targeted agents, the patient received triple chemotherapeutic drugs, including cyclophosphamide 600mg/m^2^, doxorubicin 50mg/m^2^, and vincristine 1.4mg/m^2^ (CAV), in combination with sequential re-irradiation as salvage treatment. Enhanced CT showed significant deep shrinkage in the abnormally large lesion, achieving a partial response (PR) (**Figure 1**) after 9 cycles of CAV and re-irradiation of 60 Gy in 30 fractions using intensity-modulated radiotherapy (IMRT) technique. The patient’s severe lumbar back pain improved, and neurological symptoms gradually ameliorated. She experienced increased strength in her lower limbs (graded as 3 on the MRC scale) and could walk with the aid of walking sticks. After completing 11 cycles, the patient elected to cease further maintenance chemotherapy and expired 12 months later.

## Discussion

HPC is a rare disease in the CNS, let alone in the spinal canal. The imaging features of HPC are mainly its heterogeneous vascular enhancement, large tumors with significant necrosis, internal serpentine signal voids, and bone erosion. Specific immunohistochemistry (IHC) markers such as CD34(+), CD99(+), BCL-2(+) are basis for HPC pathological diagnosis. Notably, BCL-2 was identified with a sensitivity of 96.2% (5). The 2016 WHO classified solitary fibrous tumor (SFT) and HPC as one entity and adopted its new grading system (6), and previously divided into 2 subtypes: HPC (WHO II) and anaplastic HPC (WHO III), respectively (7). Moreover, it is worth noting that, the patient had undergone a pathological transformation from WHO II (as diagnosed in previous surgical specimens) to WHO III. The later recurrence interval time was less than half of the length of the initial one, indicating the increased aggressive behavior in HPC (8, 9), which was also confirmed by the presence of a huge tumor during the recurrence. Therefore, the transformation in WHO-grade may serve as a critical indicator for short-term relapse, necessitating closer monitoring for these patients.

Surgical resection stands as the predominant treatment method for both primary and recurrent HPC in the CNS, with GTR being particularly impactful in improving overall survival (OS) (10, 11). We reviewed 13 studies (12–24) on the treatment of recurrent HPC (**Supplementary Table S1**) and found that surgery remained the cornerstone of treatment for these patients, and it was feasible whether or not adjuvant radiotherapy was added (13, 19, 22). Hence, for both newly diagnosed and repeatedly recurrent patients, the necessity of surgery should be assessed. Moreover, preoperative vascular embolization was considered to reduce the risk of intraoperative bleeding during HPC surgery (25). However, in our case, it was not used after the risk assessment, because the patient’s lesion was located in the spinal region. In the case we reported, which involved multiple recurrences and multiple surgeries, the final recurrence was judged unresectable, so systemic therapy was the most appropriate therapeutic option available.

Following the guidelines of the National Comprehensive Cancer Network (NCCN) (26), targeted agents (temozolomide plus bevacizumab or sunitinib) were deemed a generally well-tolerated and clinically beneficial regimen and the standard treatment for unresectable HPC. However, the high cost of the treatment remains a significant financial burden, particularly in developing countries. Additionally, targeted therapies did not meet expectations in patients with large tumors. In a retrospective study, patients were treated with temozolomide plus bevacizumab, and only 14.3% (2/14) achieved PR, in Response Evaluation Criteria in Solid Tumors (RECIST) criteria (27). Similarly, in another study that used sunitinib, the objective response rate (ORR) was 6.5% ((2/31) (28). These results indicated that the targeted therapies may not be sufficiently effective, particularly in cases that required rapid tumor shrinkage and symptom relief. On the other hand, the combination chemotherapy in advanced sarcoma demonstrated an impressive ORR of 88% (31/44) (29). In this case, during the third recurrence, a combination of triple chemotherapy and sequential re-irradiation proved to significantly decrease the tumor in size and alleviate symptoms. Although several regimens existed, the established standard chemotherapy regimen for HPC with recurrence or metastasis remained unclear. The combination of cyclophosphamide, doxorubicin, and vincristine (CAV) was suggested modest efficacy in patients with recurrent surgery- and radiotherapy-refractory HPC in a previous report (24). Moreover, several studies have reported that re-irradiation was used for intracranial recurrence (14, 19), but the evidence for intraspinal HPCs was scarce. It is crucial to note that re-irradiation of the spinal cord carries a high risk due to its serial organ nature, which can potentially lead to paraplegia, especially in cases of re-irradiation at the same spinal location (18). Nieder et al. suggested the feasibility of using IMRT for spinal cord re-irradiation, on the condition that the dose of each radiotherapy session did not exceed 98 Gy², the cumulative dose remained below 135.5 Gy², and the interval between treatments was over 6 months (30). Given the severe symptoms and heavy tumor burden with this massive lesion, the choice of a salvage regimen with triplet chemotherapy combined with re-irradiation was reasonable. The combination of chemotherapy with radiotherapy can have a synergistic effect (31). It was indicated that the combination of doxorubicin with radiotherapy, provided a multifaceted attack on cancer cells, targeting DNA repair mechanisms (32), increasing drug uptake (33), and enhancing cytotoxic effects (34), which could explain the high remission rates observed in clinical settings.

Overall, we have provided an exhaustive list of chemotherapy drug doses for patients and reported an instance of uncommon re-irradiation at the cauda equina with the prescription dose. Therefore, this case and its analysis hold significant value as a reference regimen for other HPC patients facing similar situations. The study has several limitations. First, targeted drugs, the standard regimen for recurring HPC, were not used in our case due to financial constraints and the relatively low response rate. Second, no additional genetic testing was performed on the patient’s pathological tissue. Finally, although the combination therapy was effective in this case, it was difficult to determine whether the outcomes were directly attributable to the chemotherapy or re-irradiation, and the effect of combination treatment needs to be validated in randomized controlled studies with larger sample sizes.

## Conclusion

In conclusion, surgical resection remains the primary treatment modality for HPC, despite its high tendency for local recurrence and the risk of metastasis. For unresectable recurrent HPC, combining chemotherapy and sequential re-irradiation may be a highly effective, and safe palliative reference regimen. The time to respond and depth of response were favorable, significantly relieving symptoms and improving the patient’s quality of life.

## Data Availability

The original contributions presented in the study are included in the article/**Supplementary Material.** Further inquiries can be directed to the corresponding author.

## References

[1] Enzinger FM Smith BH. Hemangiopericytoma. Anal 106 cases. Hum Pathol. (1976) 7:61–82. doi: 10.1016/S0046-8177(76)80006-8 1244311

[2] Combs SE Thilmann C Debus J Schulz-Ertner D. Precision radiotherapy for hemangiopericytomas of the central nervous system. Cancer. (2005) 104:2457–65. doi: 10.1002/cncr.v104:11 16222690

[3] Patro KC Palla M Kashyap R. Unusual case of metastatic intracranial hemangiopericytoma and emphasis on role of 68Ga-PSMA PET in imaging. Clin Nucl Med. (2018) 43:e331–e3. doi: 10.1097/RLU.0000000000002203 30036256

[4] Mena H Ribas JL Pezeshkpour GH Cowan DN Parisi JE. Hemangiopericytoma of the central nervous system: a review of 94 cases. Hum Pathol. (1991) 22:84–91. doi: 10.1016/0046-8177(91)90067-Y 1985083

[5] Han Y Zhang Q Yu X Han X Wang H Xu Y. Immunohistochemical detection of STAT6, CD34, CD99 and BCL-2 for diagnosing solitary fibrous tumors/hemangiopericytomas. Int J Clin Exp Pathol. (2015) 8:13166–75. PMC4680460 26722515

[6] Louis DN Perry A Reifenberger G von Deimling A Figarella-Branger D Cavenee WK. The 2016 world health organization classification of tumors of the central nervous system: a summary. Acta Neuropathol. (2016) 131:803–20. doi: 10.1007/s00401-016-1545-1 27157931

[7] Louis DN Ohgaki H Wiestler OD Cavenee WK Burger PC Jouvet A. The 2007 WHO classification of tumours of the central nervous system. Acta Neuropathol. (2007) 114:97–109. doi: 10.1007/s00401-007-0243-4 17618441 PMC1929165

[8] Wang J Zhao K Han L Jiao L Liu W Xu Y. Solitary fibrous tumor/hemangiopericytoma of spinal cord: A retrospective single-center study of 16 cases. World Neurosurg. (2019) 123:e629–e38. doi: 10.1016/j.wneu.2018.12.004 30554000

[9] Liu HG Yang AC Chen N Yang J Qiu XG Zhang JG. Hemangiopericytomas in the spine: clinical features, classification, treatment, and long-term follow-up in 26 patients. Neurosurgery. (2013) 72:16–24. doi: 10.1227/NEU.0b013e3182752f50 23147785

[10] Rana N Kim E Jaboin J Attia A. The role of adjuvant radiation in the management of solitary fibrous tumors of the central nervous system: A national cancer database analysis of 155 patients. Cureus. (2018) 10:e2656. doi: 10.7759/cureus.2656 30042907 PMC6054364

[11] Jeon SH Park SH Kim JW Park CK Paek SH Kim IH. Efficacy of adjuvant radiotherapy in the intracranial hemangiopericytoma. J Neurooncol. (2018) 137:567–73. doi: 10.1007/s11060-018-2746-3 29327171

[12] Konar S Jayan M Shukla D Bhat DI Nishant S Nandeesh BN. The risks factor of recurrence after skull base hemangiopericytoma management: A retrospective case series and review of literature. Clin Neurol Neurosurg. (2021) 208-106866. doi: 10.1016/j.clineuro.2021.106866 34388594

[13] Melone AG D’Elia A Santoro F Salvati M Delfini R Cantore G. Intracranial hemangiopericytoma—Our experience in 30 years: A series of 43 cases and review of the literature. World Neurosurgery. (2014) 81:556–62. doi: 10.1016/j.wneu.2013.11.009 24239740

[14] Hayenga HN Bishop AJ Wardak Z Sen C Mickey B. Intraspinal dissemination and local recurrence of an intracranial hemangiopericytoma. World Neurosurgery. (2019) 123:68–75. doi: 10.1016/j.wneu.2018.11.173 30503286

[15] Mickey B Hatanpaa K Ban V Flores B Patel A Barnett S. Intracranial hemangiopericytomas: recurrence, metastasis, and radiotherapy. J Neurological Surg Part B: Skull Base. (2017) 78:324–30. doi: 10.1055/s-0037-1599073 PMC5515655 28725519

[16] Veeravagu A Jiang B Patil CG Lee M Soltys SG Gibbs IC. CyberKnife stereotactic radiosurgery for recurrent, metastatic, and residual hemangiopericytomas. J Hematol Oncol. (2011) 4. doi: 10.1186/1756-8722-4-26 PMC3118387 21645367

[17] Jaber S Winer I Rasool N. Recurrent omental hemangiopericytoma: A therapeutic challenge. Case Rep Obstetrics Gynecology. (2016) 2016:1–4. doi: 10.1155/2016/2075157 PMC4819095 27088021

[18] Cohen-Inbar O Lee CC Mousavi SH Kano H Mathieu D Meola A. Stereotactic radiosurgery for intracranial hemangiopericytomas: a multicenter study. J Neurosurg. (2017) 126:744–54. doi: 10.3171/2016.1.JNS152860 27104850

[19] Rutkowski MJ Bloch O Jian BJ Chen C Sughrue ME Tihan T. Management of recurrent intracranial hemangiopericytoma. J Clin Neurosci. (2011) 18:1500–4. doi: 10.1016/j.jocn.2011.04.009 21917462

[20] Spatola C Privitera G. Recurrent intracranial hemangiopericytoma with extracranial and unusual multiple metastases: case report and review of the literature. Tumori. (2004) 90(2):265–8. doi: 10.1177/030089160409000222 15237597

[21] Spitz FR Bouvet M Pisters PW Pollock RE Feig BW. Hemangiopericytoma: a 20-year single-institution experience. Ann Surg Oncol. (1998) 5(4):350–5. doi: 10.1007/BF02303499 9641457

[22] Vignolles-Jeong J Finger G McGahan BG Beaumont TL Weber MD Wu KC. Management of recurrent giant hemangiopericytoma: illustrative cases. J Neurosurgery: Case Lessons. (2024) 7(13). 10.3171/CASE2432 PMC10971073 38531083

[23] Wang X Wang J Hu W Wang LEI Li Y. Combined therapy against recurrent and intracranial invasion of sinonasal hemangiopericytoma: A case report. Oncol Letters. (2015) 10(1):287–90. doi: 10.3892/ol.2015.3236 PMC4487133 26171016

[24] Chamberlain MC Glantz MJ. Sequential Salvage Chemotherapy for Recurrent Intracranial Hemangiopericytoma. Neurosurgery. (2008) 63(4):720–7. 10.1227/01.NEU.0000325494.69836.51 18981882

[25] Pandey M Kothari KC Patel DD. Haemangiopericytoma: current status, diagnosis and management. Eur J Surg Oncol. (1997) 23:282–5. doi: 10.1016/S0748-7983(97)90534-5 9315052

[26] von Mehren M Randall RL Benjamin RS Boles S Bui MM Conrad EU 3rd. Soft tissue sarcoma, version 2.2016, NCCN clinical practice guidelines in oncology. J Natl Compr Canc Netw. (2016) 14:758–86. doi: 10.6004/jnccn.2016.0078 27283169

[27] Park MS Patel SR Ludwig JA Trent JC Conrad CA Lazar AJ. Activity of temozolomide and bevacizumab in the treatment of locally advanced, recurrent, and metastatic hemangiopericytoma and Malignant solitary fibrous tumor. Cancer. (2011) 117:4939–47. doi: 10.1002/cncr.v117.21 PMC3135685 21480200

[28] Stacchiotti S Negri T Libertini M Palassini E Marrari A De Troia B. Sunitinib malate in solitary fibrous tumor (SFT). Ann Oncol. (2012) 23:3171–9. doi: 10.1093/annonc/mds143 22711763

[29] Pilepich MV Vietti TJ Nesbit ME Tefft M Kissane J Omer Burgert E. Radiotherapy and combination chemotherapy in advanced Ewing’s sarcoma–Intergroup study. Cancer. (1981) 47:1930–6. doi: 10.1002/1097-0142(19810415)47:8<1930::AID-CNCR2820470803>3.0.CO;2-3 7226088

[30] Nieder C Grosu AL Andratschke NH Molls M. Update of human spinal cord reirradiation tolerance based on additional data from 38 patients. Int J Radiat Oncol Biol Phys. (2006) 66:1446–9. doi: 10.1016/j.ijrobp.2006.07.1383 17084560

[31] Vokes EE Weichselbaum RR. Concomitant chemoradiotherapy: rationale and clinical experience in patients with solid tumors. J Clin Oncol. (1990) 8:911–34. doi: 10.1200/JCO.1990.8.5.911 2185342

[32] Bonner JA Lawrence TS. Doxorubicin decreases the repair of radiation-induced DNA damage. Int J Radiat Biol. (1990) 57:55–64. doi: 10.1080/09553009014550341 1967294

[33] Davies Cde L Lundstrøm LM Frengen J Eikenes L Bruland S Kaalhus O. Radiation improves the distribution and uptake of liposomal doxorubicin (caelyx) in human osteosarcoma xenografts. Cancer Res. (2004) 64:547–53. doi: 10.1158/0008-5472.CAN-03-0576 14744768

[34] Rosen G Tefft M Martinez A Cham W Murphy ML. Combination chemotherapy and radiation therapy in the treatment of metastatic osteogenic sarcoma. Cancer. (1975) 35:622–30. doi: 10.1002/1097-0142(197503)35:3<622::AID-CNCR2820350313>3.0.CO;2-C 1078640

