# Supplementary material for: Combination of triple chemotherapy and sequential re-irradiation as salvage for recurrent treatment-refractory hemangiopericytoma of extraspinal dura: a case report
Source: Front Oncol. 2024 Nov 22;14:1405755. doi: 10.3389/fonc.2024.1405755 (PMC11621096; doi:10.3389/fonc.2024.1405755)
Supplement: Supplementary file 1 [file Table1.docx]

| Table S1. Treatments for recurrent HPC | | | | | | | | | |
| --- | --- | --- | --- | --- | --- | --- | --- | --- | --- |
| Study ID | Year | Study Design | Number of patients | Location | Type of disease at presentation | Primary treatment (n) | Recurrence details | Recurrent Treatment | Survival Outcome |
| Konar(1) | 2021 | case series | 35 | Skull base | Primary | GTR (14) STR (21) | Nineteen cases had recurrences. | 13 cases underwent re-exploration and excision of the tumor. | NR |
| Melone(2) | 2014 | case series | 43 | Intracranial | Primary | GTR (19) STR (3) GTR + EBRT (11) STR + EBRT (10) | Eighteen patients had the recurrence. The median time until recurrence was 72.24 months. | All recurrent patients underwent repeat surgery. 10 patients received GTR, 2 of whom received EBRT; 8 patients received STR, 2 of whom received EBRT; 6 patients had a second recurrence and underwent repeat surgery, and 5 had a third recurrence. | The average survival period for these patients was 28.3 months. |
| Hayenga(3) | 2018 | case report | 1 | Intracranial | Primary | STR + Radiotherapy (1) | After a 3-year PFS, the patient recurred intra- and extracranially. | SRS + resection and fractionated proton beam therapy of spinal metastasis | No recurrence with 6-month follow-up. |
| Patel(4) | 2017 | article | 20 | Intracranial | Primary | GTR (13) STR (5) Unknown (2) | 11 patients had recurrence. | Two underwent repeat surgery alone, four underwent radiotherapy alone, and five underwent a combination of surgery and radiotherapy. | NR |
| Veeravagu(5) | 2011 | article | 14 (24 tumors) | Intracranial and spinal cord | Recurrent | Surgery (15) Surgery + Radiotherapy (9) | NR | CK | The total tumor control rate was 81.8%. The PFS rate was 95%, 71.5%, and 71.5% at 1, 3, and 5 years. The 5-year survival rate was 81%. |
| Jaber(6) | 2016 | case report | 1 | Omental | Primary | STR (1) | 1st recurrence: 52 months after her primary surgery 2nd recurrence: 37 months after the second surgery | 2nd and 3rd STR both without any adjuvant therapy | NR |
| Cohen-Inbar(7) | 2016 | article | 90 (133 tumors) | intracranial | Primary and Recurrent | Open cranial surgery and GKRS (90) | Tumor progression: 45.1% of tumors and 37.8% of patients; new remote intracranial tumors: 27.8% of patients; extracranial metastases: 24.4% of patients. | 35.6% of patients underwent repeat GKRS, 16.7% of patients underwent an open resection after GKRS, 12.2% of patients underwent conventional radiotherapy, and 3.3% received chemotherapy. | Post-GKRS’s local control was 54.9% of tumors and 62.2% of patients. The PFS was 89% at 2 years, 77% at 4 years, 64% at 6 years, and 54% at 8 years following a second GKRS. |
| Rutkowski(8) | 2011 | article | 14 | Intracranial | Recurrent | GTR (2) STR (2) GTR + EBRT (4) STR + EBRT (6) | NR | Eight patients underwent repeat surgical resection, of whom four received adjuvant EBRT, one received additional GKS, and one received brachytherapy. Four received GKS and two received CK. | Nine patients suffered a second recurrence at a median time of 3.5 years. Nine patients died, with a median survival of 7.9 years. |
| Spatola(9) | 2004 | case report | 1 | Intracranial | Primary | Surgery (1) | 1st recurrence: 8 years after her primary surgery 2nd recurrence: abdominal metastasis 6 months later 3rd recurrence: intracranial recurrence 19 months later | 1st recurrence: STR + Radiotherapy 2nd recurrence: radical metastasectomy + adjuvant chemotherapy (doxorubicin-carboplatin-ifosfamide) 3rd recurrence: cranial irradiation + nitrosourea-based chemotherapy | Died 9 months after the 3rd recurrence. |
| Spitz(10) | 1997 | article | 36 | Pelvis/retropefitoneum (n=14); Extremity (n=8); Epidural site (n=7); Head/neck (n=4); Thorax (n=3) | Primary (n=25); Recurrent (n=11) | GTR (28) STR (2) Chemotherapy alone (2) Radiotherapy alone (2) | Nine of the 28 patients treated with curative intent developed a local recurrence | Seven of the nine patients: surgical resection of their recurrence, and two of these patients also received adjuvant radiotherapy. | NR |
| Vignolles-Jeong(11) | 2024 | case series | 4 | Intracranial | Primary | Case 1: STR Case 2: GTR Case 3: STR Case 4: STR + Radiotherapy | Case 1: progressed 7 months later Case 2: progressed 84 months later Case 3: progressed 1 month later Case 4: progressed 39 months later | Case 1: near-total resection + Radiotherapy Case 2: GTR + Radiotherapy Case 3: GTR + Radiotherapy Case 4: GTR + Radiotherapy | Case 1: died 90 months after the initial procedure Case 2: neurologically intact Case 3: intracranially progressed 1 month later, and went under GTR + Radiotherapy again Case 4: progressed 6 months later |
| Wang(12) | 2015 | case report | 1 | Sinonasal | Recurrent | Multiple surgeries (1) | NR | Radiotherapy + Chemotherapy (Pirarubicin + Cisplatin) | No recurrence and metastasis at the 1-year follow‑up |
| Chamberlain(13) | 2008 | article | 15 | Intracranial | Recurrent | Surgery + Radiotherapy (15) | NR | Chemotherapy (CAV) | The median duration for CAV was 4 months. Nine patients received α-IFN after progression on CAV. The median duration for α-IFN was 8 months. Five received ICE after progression on α-IFN. |
| α-IFN = α-interferon; CAV = cyclophosphamide, doxorubicin, and vincristine; CK = CyberKnife stereotactic radiosurgery; EBRT = external irradiation radiotherapy; GKS/GKRS = Gamma knife radiosurgery; GTR = gross total resection; ICE = ifosfamide, cisplatin, and etoposide; OS = overall survival; PFS = progression-free survival; NR = not reported; SRS = Gamma knife stereotactic radiosurgery; STR = subtotal resection; | | | | | | | | | |

1. Konar S, Jayan M, Shukla D, Bhat DI, Nishant S, Nandeesh BN, et al. The risks factor of recurrence after skull base hemangiopericytoma management: A retrospective case series and review of literature. Clinical Neurology and Neurosurgery. 2021;208.

2. Melone AG, D'Elia A, Santoro F, Salvati M, Delfini R, Cantore G, et al. Intracranial Hemangiopericytoma—Our Experience in 30 Years: A Series of 43 Cases and Review of the Literature. World Neurosurgery. 2014;81(3-4):556-62.

3. Hayenga HN, Bishop AJ, Wardak Z, Sen C, Mickey B. Intraspinal Dissemination and Local Recurrence of an Intracranial Hemangiopericytoma. World Neurosurgery. 2019;123:68-75.

4. Mickey B, Hatanpaa K, Ban V, Flores B, Patel A, Barnett S. Intracranial Hemangiopericytomas: Recurrence, Metastasis, and Radiotherapy. Journal of Neurological Surgery Part B: Skull Base. 2017;78(04):324-30.

5. Veeravagu A, Jiang B, Patil CG, Lee M, Soltys SG, Gibbs IC, et al. CyberKnife Stereotactic Radiosurgery for Recurrent, Metastatic, and Residual Hemangiopericytomas. Journal of Hematology & Oncology. 2011;4(1).

6. Jaber S, Winer I, Rasool N. Recurrent Omental Hemangiopericytoma: A Therapeutic Challenge. Case Reports in Obstetrics and Gynecology. 2016;2016:1-4.

7. Cohen-Inbar O, Lee C-C, Mousavi SH, Kano H, Mathieu D, Meola A, et al. Stereotactic radiosurgery for intracranial hemangiopericytomas: a multicenter study. Journal of Neurosurgery. 2017;126(3):744-54.

8. Rutkowski MJ, Bloch O, Jian BJ, Chen C, Sughrue ME, Tihan T, et al. Management of recurrent intracranial hemangiopericytoma. J Clin Neurosci. 2011;18(11):1500-4.

9. Spatola C, Privitera G. Recurrent intracranial hemangiopericytoma with extracranial and unusual multiple metastases: case report and review of the literature. Tumori. 2004;90(2):265-8.

10. Spitz FR, Bouvet M, Pisters PW, Pollock RE, Feig BW. Hemangiopericytoma: a 20-year single-institution experience. Ann Surg Oncol. 1998;5(4):350-5.

11. Vignolles-Jeong J, Finger G, McGahan BG, Beaumont TL, Weber MD, Wu KC, et al. Management of recurrent giant hemangiopericytoma: illustrative cases. Journal of Neurosurgery: Case Lessons. 2024;7(13).

12. Wang X, Wang J, Hu W, Wang LEI, Li Y. Combined therapy against recurrent and intracranial invasion of sinonasal hemangiopericytoma: A case report. Oncology Letters. 2015;10(1):287-90.

13. Chamberlain MC, Glantz MJ. Sequential salvage chemotherapy for recurrent intracranial hemangiopericytoma. Neurosurgery. 2008;63(4):720-6; author reply 6-7.
